# Internet-Based Support for Cardiovascular Disease Management

**DOI:** 10.1155/2011/342582

**Published:** 2011-07-21

**Authors:** Sandra Jarvis-Selinger, Joanna Bates, Yuriko Araki, Scott A. Lear

**Affiliations:** ^1^Department of Surgery, Faculty of Medicine, The University of British Columbia, 855 West 10th Avenue, Vancouver, BC, Canada V5Z 1L7; ^2^Department of Family Practice, Faculty of Medicine, The University of British Columbia, Suite 3300, 910 West 10th Avenue, Jim Pattison Pavilion North, Vancouver General Hospital, Vancouver, BC, Canada V5Z 4E3; ^3^British Columbia Alliance on Telehealth Policy and Research, Simon Fraser University, 515 West Hastings Street, Vancouver, BC, Canada V6B 5K3; ^4^Faculty of Health Sciences, Simon Fraser University, 515 West Hastings Street, Vancouver, BC, Canada V6B 5K3

## Abstract

With significant declines in cardiovascular disease (CVD) mortality, attention has shifted to patient management. Programs designed to manage CVD require the involvement of health professionals for comanagement and patients' self-management. However, these programs are commonly limited to large urban centers, resulting in limited access for rural patients. The use of telehealth potentially overcomes geographical barriers and can improve access to care for patients. The current research explores how an Internet-based platform might facilitate collaboration among healthcare providers comanaging patients and enhance behavioural change in patients. Forty-eight participants were interviewed including: (a) patients (*n* = 12), (b) physicians (*n* = 11), (c) nurses (*n* = 13), and (d) allied health professionals (*n* = 10). The results were organized and analyzed in three central themes: (1) role of technology for CVD management, (2) challenges to technology adoption, and (3) incentives for technology adoption. Health care providers and patients supported future implementation of Internet-based technology support for CVD management.

## 1. Introduction

In recent decades, cardiovascular disease (CVD) mortality has decreased substantially in developed countries. This is a result of improved prevention and acute care. As a result of these developments and an aging population, the number of “CVD survivors” (those living with CVD) has increased. Many of these patients' disease is atherosclerotic in nature for which secondary prevention or cardiac rehabilitation programs are indicated. Effective secondary prevention of CVD is targeted to improve patient quality of life and reduce downstream morbidities. Programs designed to manage this complex chronic disease require the involvement and collaboration of physicians, nurses, allied health professionals, and multiple health services for comanagement, while also requiring knowledge uptake by patients for self-management [1]. These programs have proven effective at reducing premature mortality in patients with CVD by up to 25% over five years [2, 3]. However, they are commonly limited to large urban centres, resulting in limited access to patients in small urban, suburban, and rural communities. Indeed, patients often cite lack of geographical access as a main reason for not attending these programs [4].

The use of telehealth, defined as the use of advanced telecommunication technologies to exchange health information and health care service, has the potential to resolve these geographical inequities by providing care to patients who would otherwise not be able to receive it due to geographical barriers. Different modalities of ICTs exist for the use of telehealth and include the telephone, the Internet, and telehome monitoring. Studies using the telephone and telehome monitoring have highlighted the potential benefits of ICT use for chronic diseases. For example, the DIAL study reported a 29% reduction in hospital admissions for heart failure following a one-year telephone intervention aimed at supporting patient self-management and monitoring patient symptoms [5]. Telehome monitoring studies in a variety of chronic diseases such as heart failure, respiratory diseases, psychiatric conditions, and diabetes have also reported promising results [6–9]. In addition, patients report satisfaction with less travel, improved care, greater access to specialty care, and effective interaction of telehealth systems [10–13].

The benefits of using the Internet are far less clear. While the Internet has the benefits of being readily accessible, requiring limited resources, able to facilitate data and communication exchange, and is scalable to larger patient groups compared to telehome monitoring devices, comparatively fewer studies have studied this modality. The few studies that have, have been focused primarily on patient acceptance and feasibility, and targeted diseases other than CVD [14–17] with a few pilot studies investigating clinical outcomes [18, 19]. Despite the positive results, end user adoption is still a potential barrier to telehealth implementation [20]. In addition, it is unclear how studies in these other chronic diseases can translate to CVD in which the treatment and management of patients differ.

Our group has undertaken a number of telehealth initiatives focusing on using an Internet-based chronic disease management platform to deliver patient-focused CVD secondary prevention programs [21, 22]. We define such a platform as an interactive Internet-based tool that enables patients to monitor their symptoms and relevant physiological measures, supports care providers to tailor patient information, education, and feedback, and contains peer support features facilitated by health professionals. We also see this platform facilitating comanagement of patients, which occurs when specialists, family physicians, nurse practitioners, nutritionists, exercise specialists, and/or mental-health professionals form an interdisciplinary team in the provision of health services to manage patient illnesses or disabilities. Also titled “shared care” or “collaborative care”, comanagement varies across the health continuum—primary care, secondary care, or in the community—and varies in complexity [23]. Understanding what users, including both patients and providers, want and why they want it can guide successful telehealth implementation [8, 13]. We have conducted this investigation to better understand how an Internet-based platform can be effectively used within the context of a public provided healthcare system to deliver CVD secondary prevention programs. This research was guided by the question: how do patients and providers describe the potential role of an Internet-based platform in supporting management of patients with CVD? We were specifically interested in extending the definition provided above of an Internet-based platform for CVD management by exploring how such a platform might be helpful in facilitating collaboration among healthcare providers comanaging patients but separated by geographical distance and discipline and in enhancing behavioural change in patients.

## 2. Methods

The study received IRB approval from UBC and the health authorities involved. 

### 2.1. Context

This study took place in three areas within British Columbia, including: Vancouver, the largest city (population >2 million); a regional area 775 km north from the main city (population 83,225); rural and remote regions (seven communities ranging in size from 1,000 people to 15,281). These geographical areas were targeted because they represent the typical clinical referral route for CVD patients in the province.

### 2.2. Methodological Approach

We used qualitative methodology, employing a constructivist approach [24]. We used diffusion of innovation theory as our theoretical lens and used current telehealth literature for sensitizing concepts. The research team included a researcher in telehealth (S. Jarvis-Selinger) a kinesiologist working with CVD patients (S. A. Lear), a family physician with telehealth experience (J. Bates) and a researcher in health services and policy (Y. Araki).

### 2.3. Sampling

A purposive sample was drawn from primary and secondary care providers working in the following three settings to reflect typical referral paths for rural patients in the Northern British Columbia: (1) a tertiary care hospital in the Vancouver Metropolitan area; (2) two regional/outlying hospitals that (a) are geographically isolated from the Vancouver Metropolitan area, (b) have no in-hospital cardiac rehabilitation/heart failure management programs, and (c) routinely refer patients to the tertiary care hospital above; (3) rural communities within the service areas of the two regional hospitals. Patient participants were drawn from patients with CVD who had moved across this referral network to access care. Potential participants were identified from case files of patients referred from the two regional hospitals to the tertiary care hospital for CVD diagnosis and management and screened to ensure more than one year's experience with CVD for all participants and involvement of at least two different health professionals in different geographical settings with each patient. Experience with CVD varied widely, with urban referral cardiologists having the highest volume of CVD patients. The regional and rural practitioners from necessity have a more varied case mix and a lower overall volume of CVD patients. However, in a rural or regional context, the volume of patients is less important than the signal from the healthcare community that this is the usual caregiver of these patients in this context. All patients were drawn from rural or regional settings without access to cardiac rehabilitation programs close to home and had moved geographically across the referral network in order to access specialized care and services. 

Potential participants were either sent an email letter describing the study and requesting their participation or approached in person. Consenting individuals underwent an initial short screening interview to determine their appropriateness for participation in the study. Selection criteria limited participation to patients who were English speaking and over 19 years of age, and did not have any mental impairment. We made a particular effort to recruit female patients, but the proportion in our study is realistic, given the proportion of males to females in patients who access care at a tertiary care referral centre. A total of 48 participants were interviewed from four different groups: (a) patients (*n* = 12), (b) physicians (*n* = 11), (c) nurses (*n* = 13), and (d) allied health professionals (*n* = 10) over a period of 6 months (September 2007 to February 2008). The physician group included six general practitioners/family physicians, three cardiologists, and two internists. Cardiologists were drawn only from urban settings, as none are present in the outlying areas of British Columbia, while specialists recruited from regional settings were general internists. The nursing group included five registered nurses, six clinical nurse specialists, one community health nurse, and one nurse practitioner. The allied health professional group included five dieticians, two physical therapists, two psychologists, and one social worker. (see Table 1 for a breakdown of the demographic and geographic descriptive data). Patient participants represented a diverse range of educational backgrounds (see Table 2). 

### 2.4. Qualitative Interview Design

The researchers developed an interview framework through consultation with key health authority decision makers as well as cardiologists and internists who have experience in the comanagement of patients with CVD. The interview was designed to elicit actual experiences and resulting opinions about CVD comanagement, patient self-management, and the potential role of an internet-based platform. Specifically, our interest was in how the Internet could be used to support communication among patients, physicians, and allied healthcare professionals to improve the care of patients with CVD. We envisioned developing these programs to provide cardiac rehabilitation, support heart failure management, and allow the patients to share their progress, signs, and symptoms with a nurse case-manager in charge of their daily care. Physicians would also have the ability to interact with their patient and view progress as well, and the nurse was to communicate with the physician when needed. As themes and ideas emerged in the interviews, they were explored through probing question in subsequent interviews. Interviews were semistructured and informal, allowing the researcher to solicit further information on new areas identified by the participant. Two research assistants underwent a full day of training to standardize interview questions and probes and met regularly during data collection to discuss emerging findings and construct new questions and probes. All interviews were conducted either face-to-face or by telephone, audio-recorded, and transcribed.

### 2.5. Data Analysis

The qualitative interviews resulted in 447 pages of data. An iterative approach to data analysis was taken, employing a constant comparative method as a way to explore subjective experience [24]. This method of analysis is useful for exploring people's perspectives and developing a better understanding of their experiences [25, 26]. Such an approach pays particular attention to language and meaning, acknowledging that meaning is shaped by context [27].

Interview transcripts were analyzed in three stages by the lead author (S. Jarvis-Selinger) and a research assistant. In the first stage, all transcripts were read over repeatedly and open coding was performed to identify and label important concepts grounded in the data that were relevant to the study. Once open coding was complete, we revisited the transcripts and continued coding based on our key interview questions. This ensured that our process was open to new ideas and concepts but also relied on the work done to construct the interview questions with key stakeholders. In the second stage, the comments pertaining to these concepts were arranged in a Microsoft Excel table in order to compare the similarities and differences and group similar concepts together to establish categories and subcategories. We were particularly interested in similarities and differences across participant groups and across practice contexts. Once the table was completed, the identified categories were linked together to summarize the information into a set of common themes. 

## 3. Results

Based on the guiding research questions, the results were organized and analyzed in three central themes: (1) the role of technology for CVD management by providers and patients, (2) challenges to technology adoption, and (3) facilitators and incentives for technology adoption. Table 3 outlines the results across all theme areas, and each is discussed in turn.

### 3.1. Role of Technology for CVD Management

Healthcare providers and patients spoke about existing CVD management activities, their associated challenges, and the role that technology could play, highlighting both comanagement and self-management activities as well as communication processes. Although there was little difference in findings along geographical lines, the description of the health professionals included in the comanagement team differed according to whether they were located in a rural or urban centre. 

#### 3.1.1. Comanagement and the Role of Technology

Overall, the most commonly cited challenges to comanagement, identified across all health care provider groups, involved effective communication and the reconciliation of conflicting opinions (see Table 3). For example, physicians most often discussed communicating with teams using the discharge summary, followed by communicating via phone. All physicians stated that they did not share patient records electronically. Nurses and allied health professionals commonly cited relying on written documentation, received by mail or fax and followed by face-to-face communication where possible. Face-to-face communication was most often discussed by nurses and allied health professionals in larger clinical settings (e.g., regional and urban centres). Internet communication for comanagement was limited to the use of email by three nurses and three allied health professionals to communicate with clinicians.

From a discussion of these challenges, more than half of all health professional groups felt that the most important application of technology was to share patient health records, thus promoting timely and accurate access to information. Physicians further described the ability to electronically access patient and provider data in a timely manner as an important means of improving comanagement communication processes. As one physician noted regarding provider data, “*If we could electronically refer everything to everybody and if there was a dedicated … website that listed what services were available and physicians could go to a website to find out who's available for what service and what their mailing address, email address or what their fax number is.*” Regarding patient data, another physician observed, “*So in the end, having all those things (blood pressure, cholesterol) readily accessible in some fashion that's easy to access and interpret would certainly be of benefit to the patients and would facilitate management.*” 

#### 3.1.2. Self-Management and the Role of Technology

Physicians' suggestions for the improvement of CVD self-management included both patient- and provider-focused strategies (see Table 3). Patient-focused strategies involved online chat groups to support patient activities. Provider-focused strategies included, for example, improving resources to facilitate discussions with patients about goal setting and ensuring an available list of outpatient resources close to the patients' home for referral by providers at the time of discharge. The majority of health professionals felt that providing patients with accurate educational resources would be the best use of technology for self-management. As one nurse commented, “*I think that people could be given a website to log on to, where either it could just be simply information, where they (patients) could receive reinforcement of the printed material … So, there could be a review of that material, and that's more graphic, so you do not have to rely on literacy as much, but there could also be interactive web applications.*” 

The creation of such an Internet-based program described above was felt to be of use by all involved. For example, 21 health professionals, including all nurses and allied health professionals and five out of six general practitioners, indicated that they would use such a system. Three out of five specialists indicated that they would use such a system, while the remaining two said maybe. In addition, two-thirds of the patients stated they would be either willing to use or very interested in using aspects of an Internet-based self-management system. However, there were some concerns raised that would need to be addressed prior to implementation of an Internet-based program. Not all of these concerns were directly related to technology itself but included concerns that a program supporting patient comanagement may have the potential for leading to conflicting advice being passed onto the patient causing confusion. As well, both healthcare providers and patients were limited in their ideas about what a useful platform might consist of, citing their lack of experience with the Internet as a modality for healthcare provision as a limitation. 

### 3.2. Challenges to Technology Adoption

Health providers and patients discussed challenges related to technology adoption (see Table 3). Within these discussions, maintenance of the privacy and confidentiality of digital information was of primary concern for physicians', nurses, and allied health professionals with regard to integrating technology into CVD management. As one physician commented, “*making sure that the patient information is secure and make sure that if someone else looks at it they cannot identify the patient, having some way to identify patients as numbers*”.

For example, patients identified a lack of interest in using computers as the main barrier to technology adoption. For example on patient said, “*I'm retired and I gave all the computerization that I wanted up, that is it I do not even look at it and I will not even turn it on.*” Concerns regarding privacy issues and increased anxiety as a result of excess tracking were also articulated. As well patients felt comfortable with current methods of connecting with doctors and had a preference for face-to-face contact.

### 3.3. Facilitators and Incentives for Technology Adoption

Health professionals and patients discussed incentives that may address the identified challenges and support the use of technology for CVD management (see Table 3). Physicians identified the availability and use of other Internet-based systems such as decision support as models for development and felt that system implementation needs to take a step-wise approach that feedback should be solicited from health professionals along the implementation cycle and that physicians should be included in the decision making process. For example, physicians spoke about having high-quality resources available for patients and health professionals, as well as supporting ease of access. As one physician who spoke about ease of access commented, “*I think that getting an EMR, getting a wireless network so that I can take the computer into the office and show the patients what we are looking at has made it much easier and much better for me and the patients*”. Providers did not discuss patient involvement in this process. Unlike the more process-focused discussions from physicians, nurses, and allied health professional specifically identified time savings as the most common incentive, in addition to cost savings, the provision of training, the observation of positive patient changes, an improvement in communication, and ease of use. 

Half of the 12 patients commented on incentives that may support patient technology use. Of these, five felt that an important incentive would be to provide useful resources to patients, including patient education and easy-to-use software that could help support the tracking/monitoring of diet, exercise, and vital signs. For example, when speaking about possible technology uses, one patient talked about tracking capabilities: “*I think setting up a diet program maybe, or setting up an exercise program where you've got to tick off something everyday and say, well I've done that, you know, see what you need to do the next day… sort of setting up a routine.*” The sixth patient spoke about technological support and training as an incentive: “*I think maybe just a short little course on how to use the computer better, in all things. It would be kind of neat if I could go a couple nights a week to learn how to use it*.”

## 4. Discussion

Our study explored the perceptions of health professionals and patients about the potential role of an Internet-based platform in the management of CVD. We found that many opinions about the use of an Internet-based platform to support treatment of patients with CVD depended on the role of the interviewee (i.e., physician, nurse, allied health, or patient). These opinions did not necessarily contradict each other, but in some cases were complimentary. For example, nurses felt that physicians did not commit the time to participate to traditional comanagement opportunities, while physicians as well as allied health professionals felt that time savings would be the most effective incentive for using technology to support comanagement. However, it was noted that tensions between the health professionals' desire to provide expert advice and the patients' desire to manage their own health have the potential to disenfranchise one group or the other in a CVD management program designed for both. This would indicate that taking into account the needs and perspective of each person involved in patient management is essential to developing a “program” that can meet the needs of all involved. This feedback reflects the promotion by British Columbia's Ministry of Health Services of using a modified chronic care model adapted from the Chronic Disease Management Model described by Wagner et al. [28, 29].

Two concerns commonly expressed were the accuracy of patient self-reported data and security. These responses are consistent with findings in other studies [30, 31]. However, these are not new issues to healthcare, but it may be perceived that technology may in some way exacerbate the problems. Much of patient care relies on patients reporting their signs and symptoms to their care providers, upon which a physician will make a diagnosis. Likewise, many health providers depend on using facsimiles for communicating between one another—a method that also has concerns regarding security and privacy. Both of these issues have already been addressed in the literature and in actual program implementation [32–34]. Therefore, it is important for those developing and implementing Internet-based programs to engender the confidence in those using it.

Geographical distance created barriers for effective communication between health providers managing the same patient. It remains to be seen whether an Internet-based program can address these barriers to enable team functioning. If such a platform is to be used by providers and patients in both rural and urban settings, customization of the networks created within the management system will be important to ensure that the program is intuitive for practitioners irrespective of geographical setting and roles of team members in different settings. 

We were also struck by the degree to which both healthcare professionals and patients visualized an Internet-based platform for a CVD management system in terms of recreation of the current operations. Discharge summaries arriving late by mail, weights and exercise tracked by pen and paper, and phone discussions between family physicians and specialists could be facilitated by technology. However, there is little vision of the potential for transformation of care through technology for example, distance monitoring of patients living at home; group support for patients isolated in rural areas; 24/7 connection for patients to enable them to focus on their health beyond a 15-minute annual interaction with a health professional. Our participants cited their lack of experience with an Internet-based platform as a limitation to their understanding and ideas: this is a significant barrier to the development of a truly useful and innovative platform. The potential of the Internet to transform processes, to track discussions, or to provide asynchronous group conversation were largely ignored in favour of a recreation of current operational systems. The tension between engagement of the users in the design of a platform and the potential limitations of this engagement emerged from our findings. The consideration of a design-based research approach in the development of such platforms may mitigate this tension [35]. Strikingly, for management of a disease that depends largely on lifestyle changes made by patients, there was very little discussion from either patients or providers of how an Internet-based management system could enable behavioural change. 

A possible limitation of our study was selection bias in our participants, in that we might have recruited only participants with a keen interest in Internet-based platforms; however, we think that this was not borne out by our results. Additionally, some participants may have had too limited experience with either the Internet or other ICT to be able to consider possible uses. This is a significant limitation with our patient participants, but unavoidable with older patients, and a realistic representation of the broader community of patients. Our study took place in one province, potentially limiting its generalizability to other settings. However, a strength of our study was the sampling strategy, which sampled both diverse healthcare providers as well as patients along a usual referral route from rural communities to the regional referral centre and tertiary care urban centre, incorporating all potential users of an Internet-based platform. While the samples within each participants group were not large, qualitative research seeks to explore questions, revealing hypotheses for further testing. The multiple points of view of the research team led to rich discussion and interpretation of the findings. 

Healthcare has been notoriously slow to engage in technology that transforms business processes, and yet health is the fastest growing sector of the Internet [36, 37]. As a result of our focus on CVD, we were interacting with older patient populations for whom computers are “new”—as younger generations who have grown up with the Internet turn their interest and expectations of the Internet to health, we will see new thinking about Internet-based healthcare. In the meantime, developers must go beyond the expectations of providers and patients to develop meaningful care management systems for patients with chronic diseases. 

Our results suggest that both health care providers and patients supported the use of Internet-based technology support for CVD management, with the greatest benefit for sharing of patient data and supporting patient self-management and comanagement and with the provision of ensuring security and privacy of data. These findings are consistent with the majority of reports in the literature using Internet-based technology for CVD and other chronic disease care [38–41]. These studies have focused on the transmission and tracking of patient data and reported general patient and provider satisfaction. It is unclear what level of security of these different programs have as many articles do not go into detail on how they have addressed security and privacy concerns. However, this may be an oversight and should not be interpreted as a failure on the authors' part to address this concern. Also consistent with our findings is that many of these programs have been designed to replicate existing clinical and care duties, albeit at a distance. Again it is unclear in many studies how the systems were designed, but our results indicate that design of these systems would benefit from understanding the different perspectives of healthcare professionals and patients. Supporting change management would need to take into account how stakeholders see technology improving the current system but also moving those perspectives to the new directions that can be developed through technology. The support for implementing such systems needs to be tempered by a clear understanding of how traditional concerns (e.g., conflicting advice, data accuracy, and security) will be mitigated by such a system. 

While this study was conducted within Canada in the context of a public health care system, we believe our results to be of value and can be readily translated within other health care systems. Overall, technology supported CVD management has the potential to create positive changes to the health of patients regardless to their geographical location.

## Figures and Tables

**Table 1:** Demographic and geographic data.

Category	Participant group	Urban	Regional	Rural	Totals	Category totals
Physicians	General practitioners	0	2	4	6	11
	Cardiologists	3	0	0	3	
	Internists	0	1	1	2	

Nurses	Registered nurse	1	2	2	5	13
	Clinical nurse specialist	6	0	0	6	
	Community health	0	0	1	1	
	Nurse practitioner	1	0	0	1	

Allied health professionals	Dieticians	2	2	1	5	10
	Physical therapists	0	2	0	2	
	Psychologists	2	0	0	2	
	Social workers	1	0	0	1	

Patients	Male	0	5	6	11	12
	Female	0	1	0	1	

**Table 2:** Patient participant characteristics.

ID	Sex	Age	Education	Cardiac disease history	Recruited from
1	Male	71	High school	Coronary artery disease	Community hospital
2	Male	71	Postsecondary	Myocardial infarction	Community hospital
3	Male	71	Some postsecondary	Coronary artery disease	Community hospital
4	Male	86	High school	Coronary artery disease	Community hospital
5	Male	69	Postsecondary	Myocardial infarction	Community hospital
6	Male	78	Some postsecondary	Myocardial infarction	Community hospital
7	Male	67	High school	Myocardial infarction	Regional hospital
8	Male	56	Postsecondary	Myocardial infarction	Regional hospital
9	Male	57	Postsecondary	Atrial fibrillation and heart failure	Regional hospital
10	Male	62	Less than high school	Hypertension and atrial fibrillation	Regional hospital
11	Female	55	Less than high school	Coronary artery disease	Regional hospital
12	Male	67	No response	Myocardial infarction	Regional hospital

**Table 3:** Challenges and opportunities.

	Physicians	Nurses	Allied health	Patients
Comanagement and the role of technology: challenges				

Communication between health care workers	+	+	+	N/A
Reconciliation of conflicting opinions	+	+	+	N/A
Variation in decisions/treatments	+	+	+	N/A
Team dynamics, sorting out “who's in charge?”	+	+		N/A
Finding time to meet/time management		+	+	N/A
Geographic distances separating team members	+			N/A
Lack of physician time and buy-in		+		N/A

Comanagement and the role of technology: opportunities				

Better communication between health care workers	+	+	+	N/A
Sharing patient records	+	+	+	N/A
Timely access to accurate information	+			N/A

Self-management and the role of technology: challenges				

Lack of experience with the internet	+	+	+	+
Understanding the potential for such a platform	+	+	+	+
Potential for conflicting advice	+	+	+	

Self-management and the role of technology: opportunities				

Goal setting and tracking for patients	+	+	+	+
Accurate educational resources online for patients	+	+	+	
Creating support for patient discussions and activities and peer support	+			
Identification of available outpatient resources near patient's home	+			
Connecting with health professionals				+

Technology adoption: challenges				

Maintenance of the privacy, security, and confidentiality of digital information	+	+	+	+
Amount of time and educational support for technology uptake/tech literacy	+	+		
Infrastructure needs/lack of computers		+		+
Financial costs	+			
Accuracy of information	+			
Human resource needs		+		
Lack of interest in using computers				+
Preference for face-to-face contact				+
Increased anxiety as a result of excess tracking				+

Technology adoption: facilitators and incentives				

Provision of training		+	+	
Observation of positive patient changes		+	+	
Improvement in communication		+	+	
Time and cost saving		+	+	
Availability of high-quality resources	+			
Support ease of access	+			
Involvement in design and implementation	+			
Patient education and resources				+
Inclusion of systems to self monitor				+
Ease of use				+
